# A Rare Case of Spontaneous Splenic Rupture Secondary to Tularemia Following a Cat Bite

**DOI:** 10.7759/cureus.13218

**Published:** 2021-02-08

**Authors:** Emmanuel Fohle, Bradley A Smith, Dubert M Guerrero

**Affiliations:** 1 Internal Medicine, University of North Dakota, Fargo, USA; 2 Internal Medicine, Sanford Health, Fargo, USA; 3 Infectious Diseases, Sanford Health, Fargo, USA

**Keywords:** tularemia, spontaneous splenic rupture, cat-bite

## Abstract

Spontaneous splenic rupture is a rare but potentially life-threatening condition. More common infectious causes include infectious mononucleosis, cytomegalovirus (CMV), human immunodeficiency virus (HIV), and malaria. We present a case of a 42-year-old male who was admitted with persistent fevers, myalgia, and a recent ulcerative lesion on the base of his left thumb after a cat bite. He developed abdominal and back pains, left axillary lymphadenopathy, and near syncope. Abdominal computed tomography (CT) scan showed splenomegaly with subcapsular splenic rupture and large hematoma requiring emergent splenic embolization. Infectious work-up revealed tularemia as a cause which was successfully treated with oral doxycycline. Though not a common cat zoonoses, tularemia should be considered in a patient with splenomegaly or spontaneous splenic rupture in the setting of cat bite.

## Introduction

Splenic rupture is an uncommon occurrence in the absence of trauma. It is potentially life-threatening and often challenged by a delay in diagnosis [1]. Common infectious causes of spontaneous splenic rupture include infectious mononucleosis, cytomegalovirus (CMV), human immunodeficiency virus (HIV), and malaria [2]. In this report, we present a 42-year-old male who sustained a cat bite which manifested initially as an ulceroglandular lesion on his left forearm, axillary lymphadenopathy, and later spontaneous splenic rupture requiring emergent embolization. Tularemia presenting as spontaneous splenic rupture is a rare presentation only having been reported once in literature [3].

## Case presentation

A 42-year-old male with a past medical history of type II diabetes was admitted with left shoulder pain, abdominal and back pain, and a near syncopal event. Approximately one month prior to hospitalization, the patient was in his typical state of health when he sustained a cat bite at the base of his left thumb. He later developed a fever of 102.7^o^F and generalized body aches. He was treated with a course of amoxicillin/clavulanic acid, but within one week he returned to the clinic for soreness in his left arm, axillary lymphadenopathy, and a small ulcer at the base of his left thumb. Surface swabs of the ulcer were sent for culture but ultimately finalized with no growth. The patient was then treated with a course of trimethoprim/sulfamethoxazole, but symptoms progressed to include recurrent fevers, myalgia, mild abdominal pain, and constipation. His regimen was transitioned to doxycycline but was hospitalized after having a near syncopal event.

**Figure 1:**
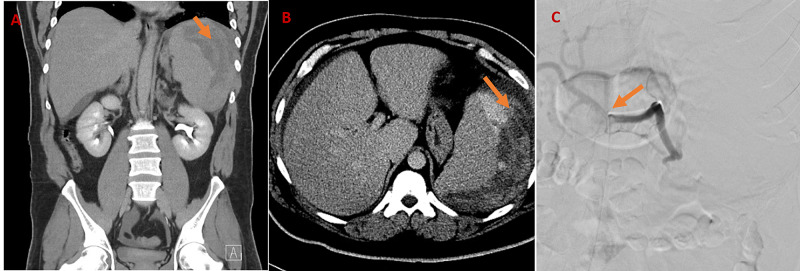
A, B: Computed tomography (CT) scan of abdomen and pelvis showing splenic hematoma; C: splenic angiogram with embolization

On admission, the patient’s vital signs were temperature of 96.8^o^F, heart rate 97 bpm, blood pressure 156/94 mmHg, respiratory rate of 21 breaths/minute on room air. Initial laboratory findings are summarized below (Table 1). Physical examination was notable for abdominal tenderness and left axillary lymphadenopathy. Abdominal computed tomography (CT) scan showed splenomegaly with subcapsular splenic rupture and large hematoma (Figures 1A-1B). The patient ultimately required an emergent splenic angiogram with embolization (Figure 1C).

**Table 1:** Laboratory findings at admission RBC: red blood cell; WBC: white blood cell; BUN: blood urea nitrogen; ALP: alkaline phosphatase; ALT: alanine aminotransferase; AST: aspartate aminotransferase; CRP: C-reactive protein; LDH: lactate dehydrogenase.

	Admission	Reference range
Hemoglobin	8.2	13-15 g/dL
RBC	3.16	4.6-6.8 x 106/mcL
WBC	17.9	3.6-10.3 x 103/mcL
Platelet	247	140-420 x 103/mcL
Absolute neutrophils	12.4	1.8-8.0 K/uL
Absolute lymphocytes	4.1	0.8-4.1 K/uL
Blood glucose	275	70-100 mg/dL
Sodium	135	135-145 mmol/L
Potassium	3.9	3.7-5.1 mmol/L
Chloride	106	96-110 mmol/L
Bicarbonate	20	22-32 mmol/L
BUN	14	6-24 mg/dL
Creatinine	0.74	0.6-1.3 mg/dL
Calcium	7.6	8.5-10.5 mg/dL
Bilirubin total	0.2	0.2-1.2 mg/dL
ALP	304	30-150 U/L
ALT	48	0-35 U/L
AST	20	0-35 U/L
CRP	177	0.0-8.0 mg/L
LDH	348	125-245 u/L
Lactic acid	0.7	0.5-2.2 mmol/L
D-dimer	1.76	≤0.49 ug/mL FEU

A broad differential was considered especially with respect to cat bites and associated diseases. The treating team later learned that the cat had succumbed to an unknown illness shortly after the bite incident. Limited testing was performed on the cat to rule out rabies, but a complete necropsy was not performed, and the remains were discarded several days prior to his hospitalization described above. The patient was vaccinated against rabies and tetanus according to guidelines. Antibiotic therapy included ampicillin/sulbactam, azithromycin, and rifampin. Because of the axillary lymphadenopathy and spontaneous splenic rupture, a broad infectious work-up was pursued but was ultimately negative in terms of Bartonella serology. Similarly, serologic work-up for Brucella, Epstein-Barr virus (EBV), and HIV were negative. Serology for endemic fungal infections including *Blastomyces dermatitidis* and *Histoplasma capsulatum* were likewise normal. The patient’s blood cultures on admission finalized without growth. Given the patient’s suboptimal response to therapy and intermittent fevers, transesophageal echocardiogram was performed but showed no radiographic evidence of endocarditis. Tularemia serology was obtained and returned positive with a titer of 1:2560. The patient’s antibiotic regimen was transitioned to doxycycline. The patient clinically improved and was eventually discharged home.

## Discussion

In this paper, we present a case of a 42-year-old male with atraumatic spontaneous splenic rupture as a complication of tularemia. This case adds another infectious agent that can be associated with spontaneous splenic rupture. 

The etiology of spontaneous splenic rupture can be categorized into six main categories including neoplastic, infectious, inflammatory, drug-induced, mechanical, and idiopathic. Various infectious agents have been associated with spontaneous splenic rupture including EBV, HIV, Plasmodium species, Salmonella, and much more [2]. Among the zoonoses, disseminated* Bartonella henselae *is well documented in the literature to present with splenic involvement as a complication [4]. However, spontaneous splenic rupture due to tularemia is rare. A previously published case report in the Annals of Internal Medicine in 1946 described a case of splenic rupture tularemia after autopsy [3]. Our review of the literature revealed no other case of tularemia induced splenic rupture in recent years.

In the United States, there are around 57 million domestic cats living in one-third of all households [5]. Cat ownership comes with its own dangers, as cats can pass on a variety of diseases to their owners. There are an estimated 400,000 cat bites each year with 66,000 hospital emergency visits each year [6]. Tularemia is a zoonotic infection caused by the facultative intracellular, gram-negative bacterium *Francisella tularensis. *It is an uncommon human pathogen most often associated with contact from either contaminated animal products or an insect or animal bite [7]. Inhalation of aerosolized droplets is associated with transmission as well. Rabbits are traditionally linked to this infection, but other sources in North America include muskrats, squirrels, voles, and beavers. Tularemia from a cat scratch or bite is rare and accounts for less than 2% of all cases of tularemia [8]. Clinical manifestations of tularemia include ulceroglandular, oculoglandular, oropharyngeal, pneumonic, typhoidal, and intestinal forms. Ulceroglandular tularemia accounts for the most common form of manifestation and is characterized by sudden onset of fever, chills, myalgia following initial infection, followed by a localized ulcer at the site of inoculation and regional lymphadenopathy [9]. The diagnosis of tularemia is primarily made by polymerase chain reaction (PCR) or detection of antibodies in serum by agglutination or enzyme-linked immunosorbent assays. Antimicrobials with efficacy for treatment include aminoglycosides, tetracyclines, quinolones, and chloramphenicol [10].

## Conclusions

Spontaneous splenic rupture is a rare complication of tularemia. Additionally, *Francisella tularensis* is not a common cat zoonoses. This case highlights the need for clinicians to broaden the differential diagnosis and include tularemia when evaluating patients with regional lymphadenopathy and splenic involvement following a cat bite.
